# Concurrent somatic KRAS mutation and germline 10q22.3-q23.2 deletion in a patient with juvenile myelomonocytic leukemia, developmental delay, and multiple malformations: a case report

**DOI:** 10.1186/s12920-018-0377-3

**Published:** 2018-07-16

**Authors:** Ruen Yao, Tingting Yu, Yufei Xu, Guoqiang Li, Lei Yin, Yunfang Zhou, Jian Wang, Zhilong Yan

**Affiliations:** 1 Department of Medical Genetics and Molecular Diagnostic Laboratory, Shanghai Children’s Medical Center, Shanghai Jiao Tong University School of Medicine, Shanghai, 200127 People’s Republic of China; 2 Institute for Pediatric Translational Medicine, Shanghai Children’s Medical Center, Shanghai Jiaotong University School of Medicine, Shanghai, 200127 People’s Republic of China; 3 Department of Internal Medicine, Shanghai Children’s Medical Center, Shanghai Jiaotong University School of Medicine, Shanghai, 200127 People’s Republic of China; 4 Rare Diseases Outpatient Clinic, Shanghai Children’s Medical Center, Shanghai Jiaotong University School of Medicine, Shanghai, 200127 People’s Republic of China; 5 Department of Pediatric Surgery, Shanghai Children’s Medical Center, Shanghai Jiaotong University School of Medicine, Shanghai, 200127 People’s Republic of China

**Keywords:** KRAS, 10q deletion, Juvenile myelomonocytic leukemia, Developmental delay, Whole-exome sequencing

## Abstract

**Background:**

The proto-oncogene KRAS performs an essential function in normal tissue signaling, and the mutation of KRAS gene is a key step in the development of many cancers. Somatic KRAS mutations are often detected in patients with solid and non-solid tumors, whereas germline KRAS mutations are implicated in patients with the Noonan syndrome, cardio-facio-cutaneous (CFC) syndrome and Costello syndrome. The deletion of chromosome 10q22.3-q23.2 is a rare cytogenetic abnormality, which often leads to distinct facial appearance and delays in speech and global development.

**Case presentation:**

Herein, we report the case of a 4-year-old boy diagnosed with juvenile myelomonocytic leukemia. The boy also had syndromic features, such as speech and motor developmental delay, multiple congenital malformations, including distinct facial features, club feet, and cryptorchidism. Using whole-exome sequencing, we identified a pathogenic mutation in KRAS [c.34G > A, p.Gly12Ser] isolated from peripheral blood DNA. Sanger sequencing confirmed the wild-type sequence in the parents and patient’s salivary cell DNA indicating its somatic state. A 7311-kb deletion in 10q22.3-q23.2 was also revealed by chromosomal microarray analysis, which was later proved as a germline de novo variant.

**Conclusion:**

Juvenile myelomonocytic leukemia in the patient was attributed to a somatic KRAS mutation, whereas the syndromic features of the patient were considered a consequence of germline chromosome 10q22.3-q23.2 deletion. Genetic testing for patients with complicated phenotypes can be valuable in detecting multiple pathogenic variants.

## Background

*KRAS*, as a member of the RAS gene family, encodes the cellular homolog of a transforming gene from the Kirsten rat sarcoma virus, which plays a vital role in normal tissue signaling, including proliferation, differentiation, and senescence. Germline or somatic mutations of *KRAS* are implicated in several human diseases like the Noonan syndrome [1], cardio-facio-cutaneous (CFC) syndrome [2], and Costello syndrome [3], as well as in different types of solid and non-solid tumor [4, 5]. *KRAS* is considered one of the most activated oncogenes, with 17% to 25 of all human tumors harboring an activating *KRAS* mutation [6].

Deletions of chromosome 10q22.3-q23.2, including that of the *BMPR1A* gene, have been associated with dysmorphic facies, developmental delay, and multiple congenital anomalies [7]. Recurrent deletions in this region derived from nonallelic homologous recombination (NAHR) between two well-defined low-copy repeats (LCRs) [8]. Larger deletions encompassing the *PTEN* gene could lead to the development of a more severe phenotype with infantile/juvenile polyposis and macrocephaly [9].

Herein, we report concurrent somatic *KRAS* mutation and germline chromosome 10q22.3-q23.2 deletion in a patient with juvenile myelomonocytic leukemia, developmental delay, and multiple congenital malformations, including distinct facial features, club feet, and cryptorchidism.

## Case presentation

A 4-year-old boy was referred to our hospital because of respiratory tract infection, splenomegaly, and thrombocytopenia. The mother was 26-year-old, and the father was 31-year-old; both were of Chinese origin, non-consanguineous and healthy. The patient had two healthy sisters. The prenatal history was unremarkable, and the patient was born via a normal delivery at term. His birth weight was 3000 g, height 50 cm, and occipitofrontal circumference 36 cm. Family history did not show any congenital malformations.

**Fig. 1:**
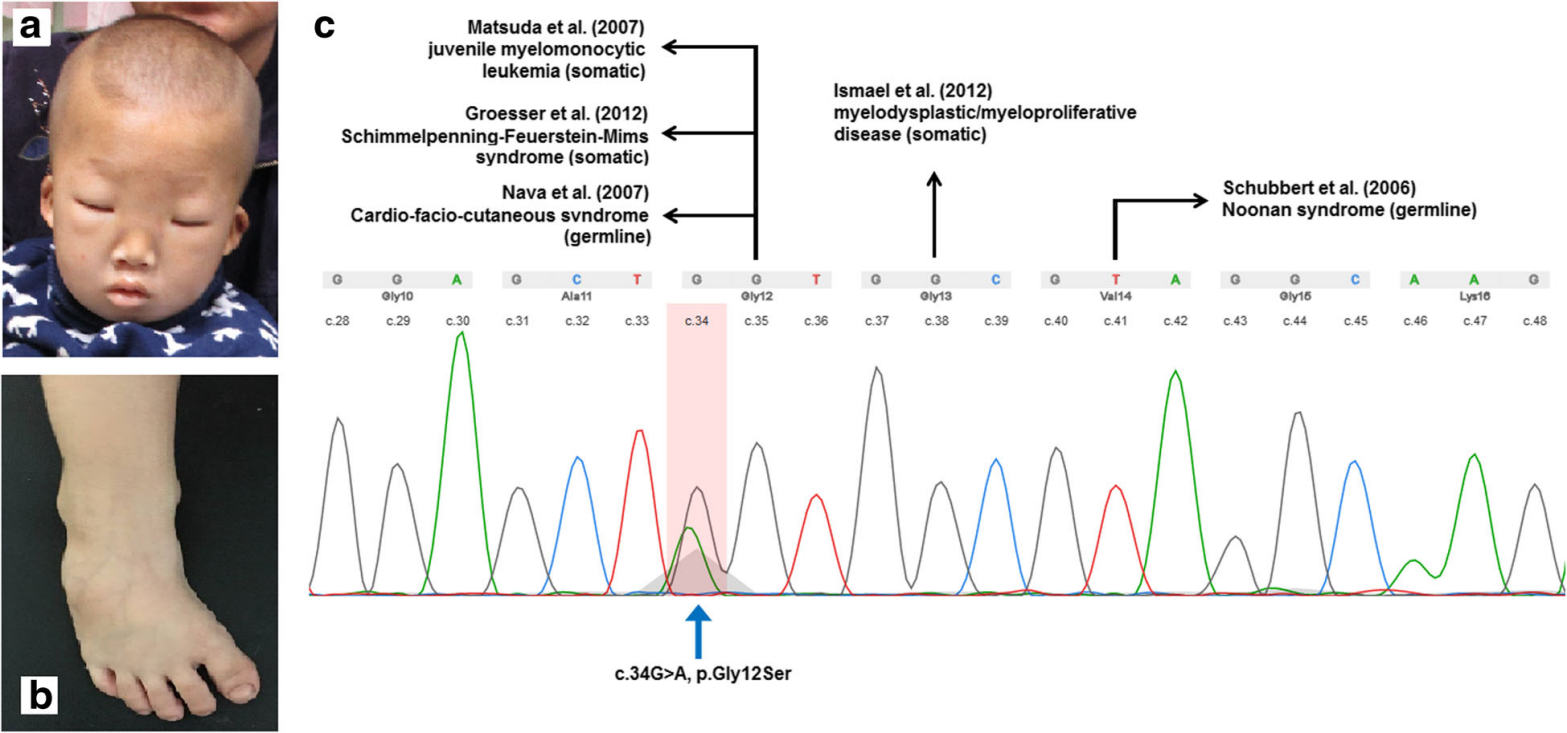
Facial feature (**a**) and club feet (**b**) of the patient. The somatic *KRAS* mutation on twelfth codon (arrow marked) of the patient and diseases caused by neighboring condon mutations are shown (**c**)

On admission, the patient showed distinct facial features, including low nasal bridge, prominent epicanthic fold, hypertelorism, and low-set ears (Fig. 1). Enlargement of the liver and spleen was also observed. Furthermore, he had congenital bilateral club feet and cryptorchidism, as well as delayed speech and motor development. A routine blood test indicated an abnormal increase of white blood cell count and hypochromic anemia. As a common symptom of JMML patients, anemia occurs when bone marrow is overcrowded by leukemia cells. Bone marrow aspiration smear revealed trilineage myelodysplasia and decreased platelet production from megakaryocyte. The diagnosis of juvenile myelomonocytic leukemia (JMML) was based on the fulfilling these criteria: (1) absence of Philadelphia chromosome or BCR/ABL fusion gene; (2) peripheral blood monocytosis > 1× 10^9^/L (peripheral blood monocyte count: 9.2×10^9^/L, peripheral blood lymphocyte count: 8.2×10^9^/L); (3) less than 20% blasts (including promonocytes) in the blood and bone marrow; (4) immature granulocytes and nucleated red cells in the peripheral blood; (5) white blood cell count > 10×10^9^/L (peripheral white blood cell count: 23.9 × 10^9^/L); (6) splenomegaly. The patient died before chemotherapy could be started and bone marrow transplantation performed due to severe infection. The CARE guidelines were followed in reporting this case.

### Whole-exome sequencing and chromosomal microarray analysis

Patient’s peripheral blood DNA was subjected to whole-exome sequencing to screen for causal variants. Briefly, 3 μg DNA was sheared to create fragments of 150–200 bp in size. An adaptor-ligated library was prepared using the paired-end sequencing library prep kit (Agilent Technologies, Santa Clara, CA, USA) and both the coding exons and flanking intronic regions were enriched with SureSelect XT Human All Exon V5 (Agilent Technologies). Then, clusters were generated by isothermal bridge amplification with an Illumina cBot station, and sequencing was performed with an Illumina HiSeq 2500 System (Illumina, San Diego, CA, USA). The Burrows Wheeler Alignment tool (BWA) v0.2.10 was employed for sequencing data alignment to the Human Reference Genome (NCBI build 37, hg 19). All data were assessed using FastQC (version 0.11.2) for quality. In addition, all single-nucleotide variants (SNVs) and indels were saved in VCF format and uploaded to Ingenuity Variant Analysis (Ingenuity Systems, Redwood City, CA, USA) for biological analysis and interpretation. Chromosomal microarray analysis (CMA) was performed using SurePrint G3 customized array (Agilent Technologies, Santa Clara, CA, USA). Previously validated platform settings were consistently utilized for CNV detection and filtering. CNVs within the size range 2–400 kb were detected via CMA and were further confirmed by manual inspection.

Using WES, we detected a heterozygous missense mutation (c.34G > A, p.Gly12Ser) in the *KRAS* gene in DNA extracted from peripheral blood of the patient, which could be categorized as pathogenic (Fig. 1). Sanger sequencing was applied to confirm the missense mutation. Further analyses of the parental blood sample and patient’s buccal swab sample revealed that the *KRAS* mutation was absent, which indicated the presence of a somatic mutation. A pathogenic deletion encompassing 7311 kb (arr[GRch37] 10q22.3q23.2 (81628905_88940359)× 1) was detected by CMA from the proband but not from his parents. The deleted region involved the OMIM genes, including *NRG3*, *CDHR1*, *RGR*, *LDB3*, *BMPR1A*, and *GLUD1*.

## Discussion and conclusions

JMML is a rare, clonal myeloproliferative/myelodysplastic disorder in children, accounting for 2–3% of the childhood hematological malignancies, which is characterized by overproduction of myelomonocytic cells that infiltrate hematopoietic and non-hematopoietic tissues [10]. Approximately 90% of the patients carry either somatic or germline mutations of *PTPN11*, *KRAS*, *NRAS*, *CBL*, or *NF1* in their leukemic cells [11]. In an earlier study, Matsuda et al. detected a somatic *KRAS* mutation in unrelated patients with JMML [12], two of the eleven patients carried the same G12S mutation as in our patient. A somatic mutation in the twelfth codon of *KRAS* was also reported in patients with nevus sebaceous tumors (G12D, G12 V) [13], the Schimmelpenning-Feuerstein-Mims syndrome (G12D) [14], RAS-associated autoimmune leukoproliferative disorder (G12D) [15], lung cancer (G12C) [16], and bladder cancer (G12R) [17]. Germline *KRAS* mutations contribute to a range of diseases or syndromes, grouped as RASopathy, which exhibit numerous overlapping phenotypic features involving multiple systems and organs due to common underlying Ras/MAPK pathway dysregulation. RASopathy-related syndromes, such as the Noonan syndrome, CFC syndrome, and Costello syndrome, can be detected in patients with germline mutations in the *KRAS* gene rather than with somatic mutations. Thus, in our study, the somatic G12S mutation detected in patient’s peripheral blood DNA, which was later confirmed to be absent from his parents and buccal swab, was considered a critical factor for the development of his JMML phenotype independent of his other symptoms.

Sporadic cases have been reported to show improvement over 2–4 year period without chemotherapy or hematopoietic cell transplantation (HCT) [12]. The majority of JMML patients ultimately require HCT for cure [18]. This option was promptly offered to any child with PTPN11, KRAS, or NF1mutated JMML and to majority of those with NRAS mutations, curing more than 50% of affected children [19].

In addition, genetic and phenotypic heterogeneity has been reported in RASopathy-related syndromes. The occurrence of a pathogenic mutation in the same codon was previously reported, but different clinical diagnoses were established in the individuals studied [2, 20]. Zenker et al. noted that patients diagnosed with Costello syndrome may later develop features of CFC syndrome [3]. Bertola et al. reported the case of a patient with K5E mutation, who was initially diagnosed with the Noonan syndrome, but later the Costello syndrome was confirmed as the final diagnosis [21]. Apart from JMML, the existing phenotypes in our patient, such as multiple malformations, including dysmorphic facial features, cryptorchidism, club feet on both sides, and developmental delay, were inconsistent with those of any of the RASopathy-related syndrome characteristics. The principal feature by which the Noonan syndrome is manifested is congenital heart defects, such as pulmonary valvular stenosis, septal defects or hypertrophic cardiomyopathy, short stature, pectus excavatum, impaired blood clotting, and characteristic facial features [1]. The CFC syndrome is characterized by distinctive facial appearance, sparse and curly scalp hair, ichthyosis, heart malformations, delayed growth, and foot abnormalities [22]. On the other hand, the Costello syndrome is featured by global delayed development, distinctive facial features, heart abnormalities, unusually flexible joints, and loose folds of extra skin, especially on the hands and feet [23]. The phenotypic discrepancy strongly suggests the possibility of another disease or syndrome caused by a second genomic event.

The CMA, which was subsequently conducted, revealed a 10q22.3-q23.2 deletion that was also categorized as pathogenic. In an earlier examination, similar deletions on the long arm of chromosome 10 were reported in six patients, four of which were with mild dysmorphic features and developmental delay [24]. A wide range of cognitive and behavioral phenotypes has been established in multiple family members having this deletion [25]. For example, Van Bon et al. has reviewed 15 cases with 10q22.3-q23 deletion or duplication [7] and found that all of the patients showed developmental delay, but their dysmorphisms and congenital anomalies differed considerably. The 10q22.3-q23.2 region is characterized by a complex set of low-copy repeats (LCRs) which can give rise to various genomic changes mediated by nonallelic homologous recombination (NAHR). The longer sequence approximately 7 Mb) between the LCRs in this region contributes to the lower frequency of recurrent 10q22.3q23 deletion than that of other recurrent deletions or duplication syndromes [25].

Two candidate genes, *BMPR1A* and *GRID1*, in the deleted region have been suggested to be related with cardiac defects. Deletion of *BMPR1A* was found to disrupt the cardiac morphogenesis in mice, resulting in various cardiac defects [26]. A previous meta-analysis of genome-wide association data proposed *GRID1* as a candidate gene responsible for the thickness of the left ventricle wall [27]. In our case, nine patients had identical breakpoints (Table 1). Cardiac defects were detected only in two patients, whereas developmental delay (9/10) and dysmorphic facial features (9/10) were manifested in most of the patients. Clinical heterogeneity might also be the reason for the low frequency of 10q22.3q23.2 deletions due to the mild phenotype established in some individuals with this deletion or miscarriages in severe individuals during pregnancy [25].

**Table 1:** Clinical Feature of Patients With Deletion in 81.6–88.9mb on Chromosome 10

Patient	1	2	3	4	5	6	7	8	9	10
Short staure*	–	–	–	–	–	–	+	+	–	–
Developmental delay	+	+	+	+	–	+	+	+	+	+
Austism	+	–	+	–	–	–	–	–	–	–
Speech delay	+	NA	+	+	–	+	–	–	+	+
OFC	P97	P97	NA	NA	NA	Macrocephaly	P10	P2	P84	P50
Cardiac defect	NA	NA	PDA	–	–	–	–	AVSD	–	–
Reference	Balciuniene et al. [2007]	Balciuniene et al. [2007]	Alliman et al. [2010]	Alliman et al. [2010]	Alliman et al. [2010]	Alliman et al. [2010]	Van Bon et al. [2011]	Van Bon et al. [2011]	Van Bon et al. [2011]	Our case
Dysmorphsims and congenital anomalies	Minor features	Ventricular structural abnormalities	Micrognathia High-arched palate Thin upper lip Wide spaced eyes Arachnodactyly Joint hyperextensibility	Hypotonia High palate Wide spaced eyes earlobe creases Prognathic mandible Rectal bleeding	Clubfeet Hearing loss Wide spaced eyes Low set ears Mild hypotonia	Small ears Wide spaced eyes Small mouth Retrognathia Mild hypotonia	Ptosis Low set small ears Hypotelorism Broad thumbs Broad halluxes Breast aplasia	Telecanthus Low set ears Hypertelorism Antervered nares Flat nasal bridge Large mouth	Low set ears Hypertelorism Radioulnar synostosis Scoliosis Kyphosis Pectus excavatum Café-au-lait spots	Clubfeet Low set ears Flat nasal bridge Wide spaced eyes Adenoid hypertrophy

*AVSD* atrial ventricular septal defect, *OFC* occipito-frontal circumference, *PDA* patent ductus arteriosus

*Short stature: < 10th centile

Molecular testing reveals the underlying genetic variant and thus substantially increases the effectiveness of diagnosis of rare diseases [28]. Furthermore, diagnostic whole-exome sequencing provides opportunities for gaining insights into the relationships between specific multi-locus genomic variations and diseases. Multiple molecular diagnoses by whole-exome sequencing were successfully performed in 4.9% of the patients in a large cohort study. The concurrent pathogenic variants in patients with multiple or ambiguous symptoms partially explained the availability of an intersectant or overlapping phenotype, which contributed to a more convincing molecular diagnosis than that based only on a single outcome [29]. Concurrent pathogenic single-nucleotide and copy number variants are more difficult to detect due to the limitation of the single testing strategy. As CNV detection were progressively optimized by analyzing whole genome exome sequencing or whole sequencing data [30, 31], genetic testing for complicated diseases, especially those with overlapping phenotype, will be more valuable while detecting multiple pathogenic variants.

## Abbreviations

CFC: Cardio-facio-cutaneous
CMA: Chromosomal microarray analysis
JMML: Juvenile myelomonocytic leukemia
LCRs: Low-copy repeats

## References

[1] Schubbert S, Zenker M, Rowe SL, Böll S, Klein C, Bollag G, van der Burgt I, Musante L, Kalscheuer V, Wehner LE (2006). Germline KRAS mutations cause Noonan syndrome. Nat Genet.

[2] Niihori T, Aoki Y, Narumi Y, Neri G, Cavé H, Verloes A, Okamoto N, Hennekam RC, Gillessen-Kaesbach G, Wieczorek D (2006). Germline KRAS and BRAF mutations in cardio-facio-cutaneous syndrome. Nat Genet.

[3] Zenker M, Lehmann K, Schulz AL, Barth H, Hansmann D, Koenig R, Korinthenberg R, Kreiss-Nachtsheim M, Meinecke P, Morlot S (2007). Expansion of the genotypic and phenotypic spectrum in patients with KRAS germline mutations. J Med Genet.

[4] Zhao S, Zhang Y, Sha K, Tang Q, Yang X, Yu C, Liu Z, Sun W, Cai L, Xu C, et al. KRAS (G12D) cooperates with AML1/ETO to initiate a mouse model mimicking human acute myeloid leukemia. Cell Physiol Biochem. 2014;33(1):78–7. 10.1159/000356651 24480914

[5] Suda K, Tomizawa K, Mitsudomi T (2010). Biological and clinical significance of KRAS mutations in lung cancer: an oncogenic driver that contrasts with EGFR mutation. Cancer Metastasis Rev.

[6] Kranenburg O (2005). The KRAS oncogene: past, present, and future. Biochim Biophys Acta.

[7] van Bon BW, Balciuniene J, Fruhman G, Nagamani SC, Broome DL, Cameron E, Martinet D, Roulet E, Jacquemont S, Beckmann JS (2011). The phenotype of recurrent 10q22q23 deletions and duplications. Eur J Hum Genet.

[8] Lupski JR, Stankiewicz P (2005). Genomic disorders: molecular mechanisms for rearrangements and conveyed phenotypes. PLoS Genet.

[9] Delnatte C, Sanlaville D, Mougenot JF, Vermeesch JR, Houdayer C, Blois MC, Genevieve D, Goulet O, Fryns JP, Jaubert F (2006). Contiguous gene deletion within chromosome arm 10q is associated with juvenile polyposis of infancy, reflecting cooperation between the BMPR1A and PTEN tumor-suppressor genes. Am J Hum Genet.

[10] Niemeyer CM, Arico M, Basso G, Biondi A, Cantu Rajnoldi A, Creutzig U, Haas O, Harbott J, Hasle H, Kerndrup G (1997). Chronic myelomonocytic leukemia in childhood: a retrospective analysis of 110 cases. Blood.

[11] Niemeyer CM. RAS diseases in children. Haematologica. 2014;99(11):1653–62. 10.3324/haematol.2014.114595 PMC4222471 25420281

[12] Matsuda K, Shimada A, Yoshida N, Ogawa A, Watanabe A, Yajima S, Iizuka S, Koike K, Yanai F, Kawasaki K (2007). Spontaneous improvement of hematologic abnormalities in patients having juvenile myelomonocytic leukemia with specific RAS mutations. Blood.

[13] Groesser L, Herschberger E, Ruetten A, Ruivenkamp C, Lopriore E, Zutt M, Langmann T, Singer S, Klingseisen L, Schneider-Brachert W (2012). Postzygotic HRAS and KRAS mutations cause nevus sebaceous and Schimmelpenning syndrome. Nat Genet.

[14] Rijntjes-jacobs E, Lopriore E, Steggerda SJ, Kant SG, Walther FJ (2010). Discordance for Schimmelpenning-Feuerstein-Mims syndrome in monochorionic twins supports the concept of a postzygotic mutation. Am J Med Genet A.

[15] Niemela JE, Lu L, Fleisher TA, Davis J, Caminha I, Natter M, Beer LA, Dowdell KC, Pittaluga S, Raffeld M (2011). Somatic KRAS mutations associated with a human nonmalignant syndrome of autoimmunity and abnormal leukocyte homeostasis. Blood.

[16] Nakano H, Yamamoto F, Neville C, Evans D, Mizuno T, Perucho M (1984). Isolation of transforming sequences of two human lung carcinomas: structural and functional analysis of the activated c-K-ras oncogenes. Proc Natl Acad Sci U S A.

[17] Santos E, Martin-Zanca D, Reddy EP, Pierotti MA, Della Porta G, Barbacid M (1984). Malignant activation of a K-ras oncogene in lung carcinoma but not in normal tissue of the same patient. Science.

[18] Dvorak CC, Loh ML (2014). Juvenile myelomonocytic leukemia: molecular pathogenesis informs current approaches to therapy and hematopoietic cell transplantation. Front Pediatr.

[19] Locatelli F, Niemeyer CM (2015). How I treat juvenile myelomonocytic leukemia. Blood.

[20] Kratz CP, Zampino G, Kriek M, Kant SG, Leoni C, Pantaleoni F, Oudesluys-Murphy AM, Di Rocco C, Kloska SP, Tartaglia M (2009). Craniosynostosis in patients with Noonan syndrome caused by germline KRAS mutations. Am J Med Genet A.

[21] Bertola DR, Pereira AC, Brasil AS, Albano LM, Kim CA, Krieger JE (2007). Further evidence of genetic heterogeneity in Costello syndrome: involvement of the KRAS gene. J Hum Genet.

[22] Nyström AM, Ekvall S, Berglund E, Björkqvist M, Braathen G, Duchen K, Enell H, Holmberg E, Holmlund U, Olsson-Engman M (2008). Noonan and cardio-facio-cutaneous syndromes: two clinically and genetically overlapping disorders. J Med Genet.

[23] Rosty C, Young JP, Walsh MD, Clendenning M, Walters RJ, Pearson S, Pavluk E, Nagler B, Pakenas D, Jass JR (2013). Colorectal carcinomas with KRAS mutation are associated with distinctive morphological and molecular features. Mod Pathol.

[24] Alliman S, Coppinger J, Marcadier J, Thiese H, Brock P, Shafer S, Weaver C, Asamoah A, Leppig K, Dyack S (2010). Clinical and molecular characterization of individuals with recurrent genomic disorder at 10q22.3q23.2. Clin Genet.

[25] Balciuniene J, Feng N, Iyadurai K, Hirsch B, Charnas L, Bill BR, Easterday MC, Staaf J, Oseth L, Czapansky-Beilman D (2007). Recurrent 10q22-q23 deletions : a genomic disorder on 10q associated with cognitive and behavioral abnormalities. Am J Hum Genet.

[26] Gaussin V, Van de Putte T, Mishina Y, Hanks MC, Zwijsen A, Huylebroeck D, Behringer RR, Schneider MD (2002). Endocardial cushion and myocardial defects after cardiac myocyte-specific conditional deletion of the bone morphogenetic protein receptor ALK3. Proc Natl Acad Sci U S A.

[27] Vasan RS, Glazer NL, Felix JF, Lieb W, Wild PS, Felix SB, Watzinger N, Larson MG, Smith NL, Dehghan A (2009). Genetic variants associated with cardiac structure and function: a meta-analysis and replication of genome-wide association data. JAMA.

[28] Jamuar SS, Tan E (2015). Clinical application of next-generation sequencing for Mendelian diseases. Hum Genomics.

[29] Posey JE, Harel T, Liu P, Rosenfeld JA, James RA, Coban Akdemir ZH, Walkiewicz M, Bi W, Xiao R, Ding Y (2017). Resolution of disease phenotypes resulting from multilocus genomic variation. N Engl J Med.

[30] Tan R, Wang Y, Kleinstein SE, Liu Y, Zhu X, Guo H, Jiang Q, Allen AS, Zhu M (2014). An evaluation of copy number variation detection tools from whole-exome sequencing data. Hum Mutat.

[31] Yao R, Zhang C, Yu T, Li N, Hu X, Wang X, Wang J, Shen Y (2017). Evaluation of three read-depth based CNV detection tools using whole-exome sequencing data. Mol Cytogenet.

